# Variability in Syndesmotic Screw Angles During Ankle Fracture Fixation: Insights From Postoperative CT Analysis

**DOI:** 10.7759/cureus.101648

**Published:** 2026-01-15

**Authors:** Tal Shachar, Oz Cohen, Omer Marom, Geva Sarrabia, Dor Dan, Nadav Haddad, David Segal, Ezequiel Palmanovich, Eyal Yaacobi, Nissim Ohana

**Affiliations:** 1 Orthopedics, Meir Medical Center, Kfar Saba, ISR; 2 Orthopedic Surgery, Meir Medical Center, Kfar Saba, ISR

**Keywords:** ankle fracture, computed tomography, orthopedic outcomes, radiographic assessment, screw angle, screw trajectory, surgical technique, syndesmotic reduction, syndesmotic screw fixation, tibiofibular diastasis

## Abstract

Background

Syndesmotic screw fixation is a widely used technique for stabilizing ankle fractures with syndesmotic disruption. While current guidelines recommend screw insertion at an angle of 30°-45° relative to the anterior foot, limited evidence supports whether these angles are routinely achieved in clinical settings or are necessary for optimal outcomes. This study aimed to assess the angles actually obtained in practice and to identify radiographic factors associated with successful syndesmotic reduction.

Methods

This retrospective study evaluated 100 patients treated with syndesmotic screw fixation for ankle fractures at a secondary referral hospital. Postoperative CT scans were used to assess screw angle, tibiofibular distances, and the quality of syndesmotic reduction. All angle measurements were taken in the neutral ankle position using the second metatarsal head as a reference. Associations between screw trajectory and reduction parameters were analyzed using IBM SPSS Statistics for Windows, Version 29.0.2.0 (Released 2022; IBM Corp., Armonk, NY, USA) and WinPEPI software.

Results

Of the 100 patients, 72% achieved a good syndesmotic reduction. Demographic variables (age, sex), side of injury, injury mechanism, and number of screws showed no significant impact on reduction quality. However, posterior screw placement was significantly associated with poor reduction (p = 0.013). Patients with poor reductions demonstrated significantly higher anterior and posterior tibiofibular distances, greater diastasis, and steeper screw angles (p < 0.001).

Conclusions

There is a notable discrepancy between the recommended and actual screw angles achieved in practice. Lower angles (~15°) were associated with favorable reductions, challenging the necessity of adhering to the 30°-45° guideline. Further prospective studies are needed to refine surgical recommendations based on clinical outcomes.

## Introduction

Ankle fractures can occur through various mechanisms, most commonly resulting from sudden and forceful twisting, bending, or direct impact to the joint [1]. Nonetheless, it is important to recognize that some ankle fractures may arise from minimal trauma, particularly in elderly individuals [2].

The incidence of ankle fractures in the adult population is approximately 187 per 100,000, with the highest occurrence observed among males aged 15-24 years and females aged 75-84 years [3,4]. These injuries often result in Denis-Weber Type B or C fractures, which are frequently accompanied by syndesmotic disruption, reported in up to 40% of Type B fractures and as many as 80% of Type C fractures [5].

Surgical intervention is typically warranted in such cases, depending on the severity and anatomical location of the injury. A commonly employed surgical technique is syndesmotic screw fixation, which aims to stabilize the distal tibiofibular joint. This method has been reported to yield success rates exceeding 90% [6]. It is frequently performed in conjunction with ancillary procedures such as ankle arthroscopy or ligament repair, followed by a period of immobilization and structured rehabilitation to facilitate functional recovery [7].

Despite its widespread use, the optimal strategy for syndesmotic fixation remains a subject of ongoing debate. Key areas of contention include the number, diameter, angle, and length of the screw, as well as the necessity and timing of screw removal [8].

The principal goal of screw placement is to achieve stable fixation of the syndesmosis while maintaining some degree of physiologic motion at the ankle joint. Among the technical variables, the angle of screw insertion is considered particularly influential in determining the quality of syndesmotic reduction [7].

Historically, it has been recommended that the syndesmotic screw be inserted at an angle of approximately 30°-45° relative to the horizontal plane and around 15° to the sagittal plane [9-11]. However, emerging evidence suggests that favorable outcomes may still be attained even when screws are inserted at angles deviating from these traditional guidelines [12,13].

The primary objective of this study was to determine the association between the actual angle of syndesmotic screw insertion and syndesmotic reduction quality as assessed on postoperative CT. Secondary objectives were to characterize the real-world variability in achieved screw insertion angles under routine surgical conditions and to explore additional radiographic predictors of syndesmotic malreduction. This study was designed as a hypothesis-driven investigation evaluating whether deviations from recommended screw trajectories are associated with inferior reduction quality.

## Materials and methods

Study design and setting

A retrospective study was conducted in the Orthopedic Surgery Department of Meir Medical Center, a secondary referral hospital, between January 2021 and December 2022. The institutional review board approved the study protocol, and the investigation adhered to the principles of the Declaration of Helsinki (1964) and its subsequent amendments. Because of the retrospective nature of the study, the requirement for informed consent was waived.

Participants

The cohort comprised 100 patients. Inclusion criteria were age ≥18 years and patients who presented with ankle fractures and underwent syndesmotic screw fixation. No clinical outcome data were collected. Exclusion criteria included age <18 years, congenital or acquired deformities likely to alter foot alignment, and absence of a postoperative CT scan.

Eligible patients were identified through a structured electronic query of the institutional medical record system using ICD-10 diagnostic codes for ankle fractures and procedural codes for syndesmotic fixation. All potentially eligible cases were manually screened by two investigators to confirm the presence of syndesmotic screw fixation and the availability of postoperative CT imaging.

Postoperative CT scans were acquired using a standardized institutional protocol, with patients positioned supine and the ankle in neutral alignment under non-weight-bearing conditions. Imaging was performed using a multidetector CT scanner with a slice thickness of approximately 0.6-1.25 mm and standard bone reconstruction algorithms.

All radiographic measurements were independently performed by two senior orthopedic surgeons who were blinded to each other’s measurements and to clinical classification. Interobserver reliability was assessed using the intraclass correlation coefficient (two-way random-effects model, absolute agreement). Reduction quality was classified based on established CT-based syndesmotic alignment parameters, with good reduction defined as restoration of normal distal tibiofibular congruency within accepted anatomic thresholds for anterior tibiofibular distance (ATFD), posterior tibiofibular distance (PTFD), and tibiofibular clear space (TFCS). Poor reduction was defined as persistent malalignment exceeding these limits.

Operative variables such as screw diameter, number of cortices engaged, and fixation technique were not standardized and were determined according to individual surgeon preference; therefore, these parameters were not included as quantitative covariates in the primary analysis.

Data management and radiographic measurements

**Figure 1:**
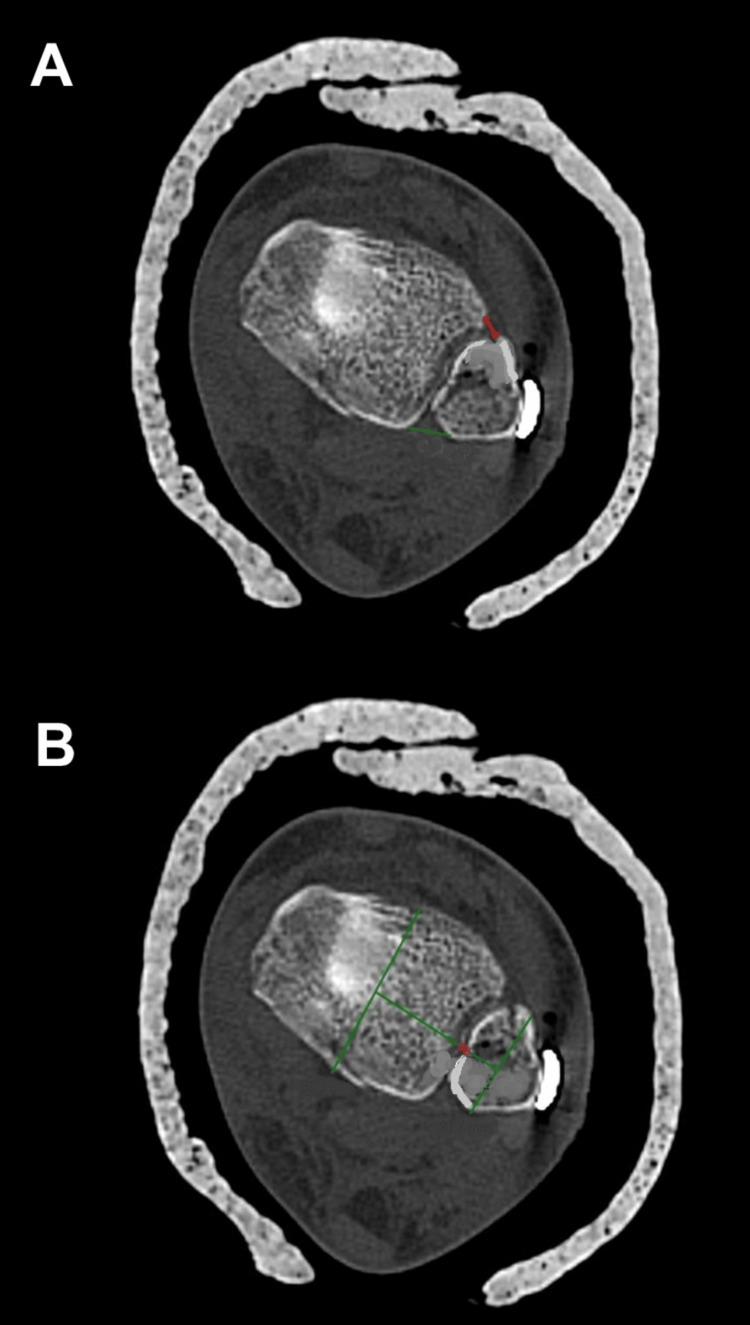
Measurements evaluating the diastasis between the tibia and fibula proximal to the tibial plafond (A) ATFD, measured as the shortest distance between the anterior cortex of the fibula and the corresponding anterior aspect of the tibial incisura. PTFD is defined as the shortest distance between the posterior cortex of the fibula and the posterior margin of the tibial incisura. (B) Tibial incisura length line, defined as the linear distance between the anterior and posterior margins of the tibial incisura. ATFD, anterior tibiofibular distance; PFTD, posterior tibiofibular distance

Records were screened in several stages to confirm the total number of surgically managed ankle fractures and to identify those treated with syndesmotic screws. Postoperative CT scans were reviewed by a single author, who measured the following parameters: ATFD, PTFD, TFCS, and screw insertion angle (Figure 1).

ATFD is defined as the shortest linear distance between the anterior cortex of the fibula and the anterior margin of the tibial incisura, measured on an axial CT slice obtained 10 mm proximal to the tibial plafond. PTFD is defined as the shortest linear distance between the posterior cortex of the fibula and the posterior margin of the tibial incisura, measured at the same standardized axial level. Diastasis is defined as pathologic widening of the distal tibiofibular joint, quantified on axial CT imaging using syndesmotic parameters (ATFD, PTFD, and/or TFCS). Increased values indicate loss of normal tibiofibular congruency and syndesmotic instability.

The screw insertion angle is defined as the angular orientation of the syndesmotic screw relative to a predefined tibial reference axis on axial CT imaging, representing the deviation of the actual screw trajectory from the ideal or intended trajectory across the distal tibiofibular joint.

Additional variables extracted from the electronic medical record included demographics, admission date, surgery date, length of hospital stay, and mechanism of injury.

**Figure 2:**
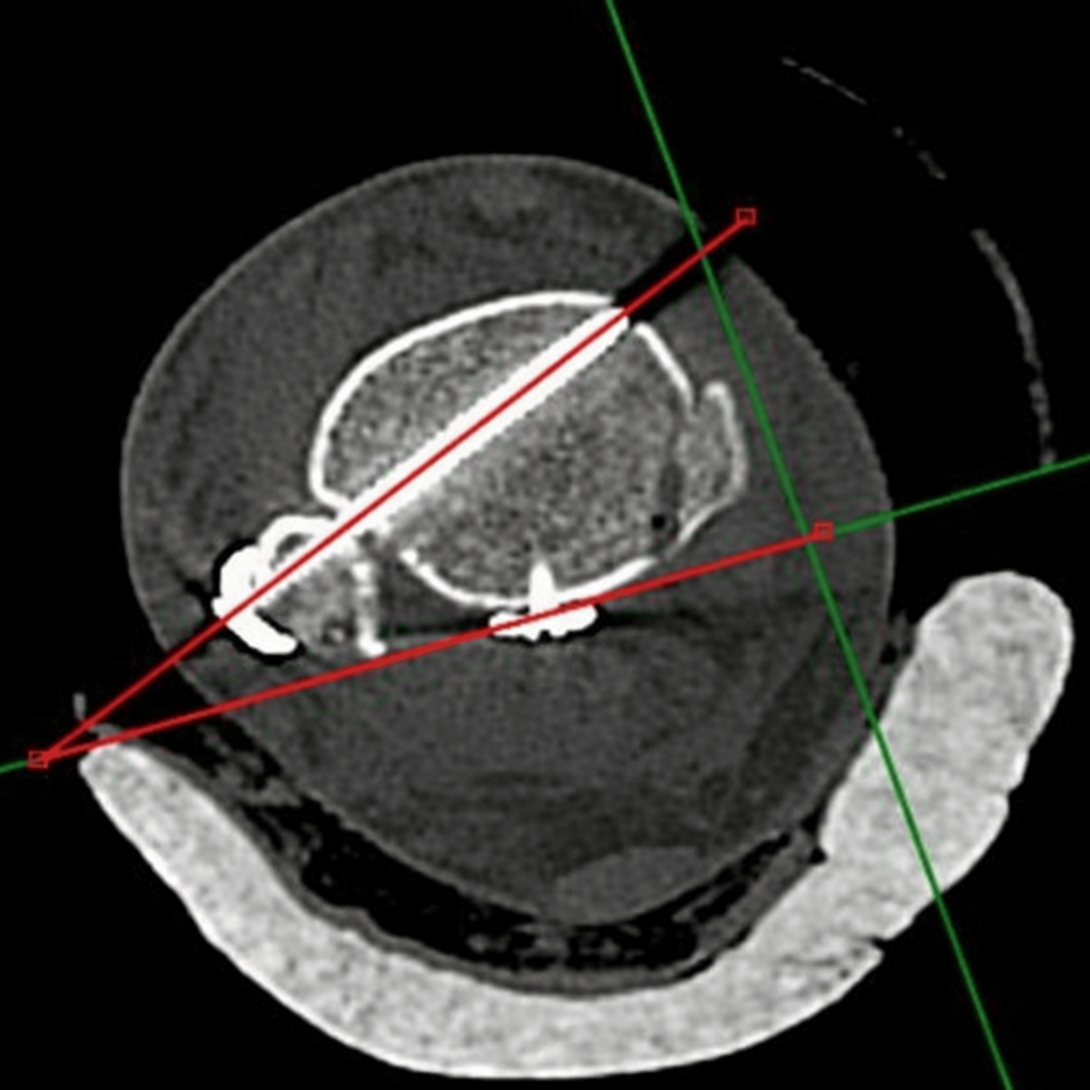
Measurement of the angle of syndesmotic screw fixation

Angle measurements were performed with the ankle in a neutral position and the second toe oriented anteriorly (Figure 2). A longitudinal reference line was drawn through the second proximal phalanx toward the ankle joint, and a perpendicular line to this reference was then constructed. A third line was drawn from the apex of the fibular cortex through the center of the tibia. The angle between the second and third lines defined the ideal trajectory for syndesmotic screw fixation [12].

Statistical analysis

Continuous variables are presented as means ± SDs, whereas categorical variables are expressed as counts and percentages. Between-group differences for continuous data were assessed using the independent-samples Student’s t-test, and categorical data were analyzed using Fisher’s exact test. Effect sizes were reported as Cohen’s d (≈0.2 small, 0.5 medium, 0.8 large). A two-sided α of 0.05 and β of 0.20 (power = 80%) were adopted for statistical significance. Analyses were performed using IBM SPSS Statistics for Windows, Version 29.0.2.0 (Released 2022; IBM Corp., Armonk, NY, USA).

Post hoc power analysis

Given the available sample size, the study had 80% power to detect a 9.8° difference in screw trajectory at a 95% confidence level. Detection of the observed 4° difference would require a substantially larger cohort, estimated at approximately four times the present sample size (124 patients with good reduction and 96 with poor reduction).

Good reduction was defined as restoration of normal distal tibiofibular alignment on postoperative CT imaging, with ATFD, PTFD, and TFCS values within accepted anatomic limits. Poor reduction was defined as persistent syndesmotic malreduction on CT imaging, characterized by abnormal widening or asymmetry of one or more syndesmotic parameters.

## Results

Of the 100 included patients (mean ± SD age, 49.6 ± 16.8 years), 61 were male (61%), and 23 (23%) presented with a right-sided injury (Table 1). Comparison between the good-reduction (n = 72) and poor-reduction (n = 28) cohorts revealed no significant differences in age (49.8 ± 17.7 vs 49.1 ± 14.8 years; p = 0.914), sex distribution (56.9% vs 71.4% male; p = 0.369), or laterality of injury (p = 0.970).

**Table 1:** Descriptive statistics of the study sample Continuous variables are presented as mean ± SD and were compared using the independent-samples t-test. Categorical variables are presented as numbers (percentages) and were compared using the χ² test or Fisher’s exact test, as appropriate. ATFD, anterior tibiofibular distance; MVA, motor vehicle accident; PTFD, posterior tibiofibular distance

Variable	Good reduction (n = 72, 72%)	Poor reduction (n = 28, 28%)	Total (n = 100, 100%)	Test statistic	p-Value
Age (years)	49.75 ± 17.74	49.12 ± 14.78	49.58 ± 16.81	t = 0.11	0.914
Side, right	17 (23.6%)	6 (21.4%)	23 (23%)	χ² = 0.001	0.97
Sex, male	41 (56.9%)	20 (71.4%)	61 (61%)	χ² = 0.81	0.369
Injury mechanism				χ² = 0.64	0.892
Fall	39 (54.2%)	14 (50%)	53 (53%)		
MVA	13 (18.1%)	7 (25%)	20 (20%)		
Sports	9 (12.5%)	3 (10.7%)	12 (12%)		
Other trauma	11 (15.3%)	4 (14.3%)	15 (15%)		
Screws				χ² = 1.84	0.175
One	55 (76.4%)	25 (89.3%)	80 (80%)		
Two	17 (23.6%)	3 (10.7%)	20 (20%)		
Screw position				χ² = 8.61	0.013
Anterior	37 (51.4%)	19 (67.9%)	56 (56%)		
Middle	34 (47.2%)	6 (21.4%)	40 (40%)		
Posterior	1 (1.4%)	3 (10.7%)	4 (4%)		
ATFD (mm)	1.75 ± 0.28	3.34 ± 1.06	2.20 ± 0.94	t = -9.27	<0.001
PTFD (mm)	3.33 ± 0.43	4.98 ± 0.84	3.79 ± 0.94	t = -10.10	<0.001
Diastasis (mm)	1.80 ± 0.39	3.53 ± 0.87	2.28 ± 0.96	t = -11.40	<0.001
Screw angle (°)	15.00 ± 8.70	22.94 ± 12.43	17.94 ± 10.36	t = -3.24	0.002

Good reduction was achieved in 72 patients (72%), whereas 28 patients (28%) demonstrated suboptimal reduction. The mechanism of injury was similar between groups, with falls accounting for just over half of cases, followed by motor vehicle accidents, sports injuries, and other trauma (p = 0.892). The number of syndesmotic screws (one vs. two) likewise did not influence reduction quality (p = 0.175).

Screw position, however, showed a significant association with reduction outcome (p = 0.013). Posterior screw placement was more common in the poor-reduction group (10.7%) than in the good-reduction group (1.4%), whereas anterior and middle positions predominated among well-reduced cases.

Radiographic metrics demonstrated clinically important differences. The poor-reduction group had greater ATFD (3.34 ± 1.06 mm vs. 1.75 ± 0.28 mm), PFTD (4.98 ± 0.84 mm vs. 3.33 ± 0.43 mm), and syndesmotic diastasis (3.53 ± 0.87 mm vs. 1.80 ± 0.39 mm), all p < 0.001. The mean screw angle was also larger in poorly reduced ankles (22.94 ± 12.43° vs. 15.00 ± 8.70°; p = 0.002).

Together, these findings highlight screw trajectory and postoperative syndesmotic alignment, rather than baseline demographics, injury pattern, or screw number, as key determinants of reduction quality.

## Discussion

In this study, we aimed to evaluate the optimal angle of syndesmotic screw placement in ankle fractures and to assess whether the angles recommended in the literature correspond to those achieved in clinical practice. Additionally, we sought to identify other factors that may influence successful surgical outcomes.

Achieving anatomical reduction in ankle fractures is critical for restoring normal joint alignment, promoting healing, ensuring stability, minimizing long-term complications, and optimizing functional recovery [14]. It is widely accepted that precise screw placement plays a key role in syndesmotic fixation; however, current literature offers limited guidance on the optimal screw trajectory.

Our findings indicate that, with the ankle in a neutral position and the second toe aligned anteriorly, the mean ideal screw angle was 15.5°, which is lower than the conventionally recommended 30°-45° range. This discrepancy suggests that, in real-world surgical settings, achieving the recommended angles may not always be practical or necessary.

Comparison with previous studies

Previous studies have largely focused on the biomechanical properties of syndesmotic fixation rather than on the precise angles at which screws should be placed [15-17]. While the 30°-45° recommendation has been widely cited, it is not always clearly defined [18,19]. Some studies describe this range in reference to the longitudinal axis of the tibia, while others relate it to the anterior position of the foot [18,20]. However, this definition remains ambiguous, as proper alignment should account for the second metatarsal head being positioned truly anteriorly to ensure accurate angulation.

Interestingly, our study’s lower mean angle of 15.5° raises questions about whether the traditionally recommended angulation is necessary for optimal outcomes. If the lower angles achieved intraoperatively still provide sufficient syndesmotic stability, rigid adherence to a higher angle range may not be clinically justified.

Clinical and surgical implications

The observed lower mean screw angle in this cohort may have potential clinical relevance; however, these findings should be interpreted cautiously. While biomechanical studies suggest that an ideal screw trajectory should balance rigid fixation with allowance for physiological micromotion of the syndesmosis, our data demonstrate an association rather than a causal relationship between screw angle and radiographic reduction quality. A more perpendicular trajectory (closer to 15°) may be compatible with adequate fixation, but the present study does not establish that such angles are inherently superior, nor does it permit conclusions regarding implant longevity, pain, or functional recovery [21].

From a practical surgical perspective, achieving the traditionally recommended 30°-45° angulation may be technically challenging because of anatomic constraints, intraoperative positioning, soft tissue swelling, or fracture complexity. Fluoroscopic guidance is commonly used for screw placement, yet precise control of angulation remains difficult in routine practice. Our findings suggest that lower angles may be more commonly achieved under real-world conditions; however, this observation should be regarded as hypothesis-generating rather than practice-changing. Prospective studies incorporating standardized surgical protocols and patient-reported outcomes are required to determine whether these radiographic associations translate into clinically meaningful benefits.

Study strengths and limitations

A major strength of this study is the use of CT-based measurements, which provide an objective and accurate assessment of screw trajectory. Unlike plain radiographs, CT imaging allows for precise three-dimensional evaluation of screw positioning, ensuring reliable data collection.

However, this study has several limitations that should be considered when interpreting the findings. First, the retrospective design inherently introduces the potential for selection bias and unmeasured confounding. Inclusion was restricted to patients who underwent postoperative CT imaging, which may not represent the full spectrum of surgically treated ankle fractures and could bias the cohort toward more complex or equivocal cases.

Second, although radiographic measurements were performed by experienced surgeons, the assessments were not originally designed with a fully standardized, blinded protocol, and interobserver reliability was not prospectively incorporated into the study design, which may affect the reproducibility of the measurements.

Third, key operative variables, including screw diameter, number of cortices engaged, reduction technique, and surgeon experience, were not standardized and were determined according to individual surgeon preference. These factors were therefore not controlled analytically and may act as important confounders influencing the reduction quality.

Furthermore, this study focused exclusively on radiographic parameters of syndesmotic alignment and did not include functional, patient-reported, or long-term clinical outcomes. As a result, conclusions regarding the “effectiveness” of specific screw angles should be interpreted cautiously, as improved radiographic reduction does not necessarily translate into superior clinical outcomes.

Finally, the single-center nature of the study may limit the generalizability of the findings to other institutions with different surgical techniques, imaging protocols, or patient populations.

Future directions

Further research is needed to assess whether screw angles closer to 15°-20° provide comparable or superior clinical outcomes compared with the conventionally recommended 30°-45° range. Prospective studies with larger sample sizes and long-term follow-up should evaluate the impact of screw angle on syndesmotic stability, healing rates, implant failure, and functional recovery. Additionally, biomechanical studies could explore whether a more perpendicular trajectory influences screw pullout strength, load distribution, and postoperative complications.

## Conclusions

Our findings demonstrate a discrepancy between commonly cited theoretical recommendations of a 30°-45° syndesmotic screw trajectory and real-world surgical practice. In this cohort, a mean insertion angle of approximately 15.5° was associated with acceptable radiographic reduction quality; however, this association should not be interpreted as evidence of superiority or clinical effectiveness. Rather, the data suggest that surgeons may commonly achieve lower angles in routine operative settings, likely reflecting anatomic constraints and technical considerations.

Importantly, this study evaluates radiographic alignment rather than functional or patient-centered outcomes and therefore cannot support practice-changing recommendations regarding optimal screw trajectory. Future prospective studies incorporating standardized surgical techniques, biomechanical validation, and long-term clinical outcomes are needed to determine whether specific angular ranges, including lower trajectories around 15°, translate into meaningful clinical benefit.
